# Grassland Saline-Alkaline Degradation-Induced Excessive Iron and Sodium Intake Potentially Increases the Transmission Risk of Fecal Pathogenic Bacteria in Cattle

**DOI:** 10.3390/ani15233484

**Published:** 2025-12-03

**Authors:** Yizhen Wang, Bingnan Gao, Guangming Ma, Man Xu, Yu Zhou, Xin Jiang

**Affiliations:** Jilin Songnen Grassland Ecosystem National Observation and Research Station, Key Laboratory of Vegetation Ecology of the Ministry of Education, Institute of Grassland Science, Northeast Normal University, Changchun 130024, China

**Keywords:** grassland saline-alkaline degradation, herbivore grazing, pathogenic bacteria, iron intake, sodium intake

## Abstract

The environmental transmission of pathogens from domesticated ruminants presents a significant risk to the well-being of both humans and animals. However, the risk of pathogen release from grazing ruminants in natural grasslands has received comparatively little attention. The threat of pathogen release from grazing ruminants may be increasing owing to grasslands in arid and semi-arid regions experiencing prevalent saline-alkaline degradation. Therefore, further risk assessment of pathogen release driven by saline-alkaline degradation is warranted. Here, we investigated the impact of saline-alkaline degradation in grasslands on the fecal pathogenic bacteria of domestic ruminant cattle by comparing cattle subjected to long-term grazing on undegraded (UG) and severe saline-alkaline degraded (SG) grasslands. Our results showed that the relative abundance of potential pathogenic bacteria including *Streptococcus*, *Mogibacterium*, and *Alistipes* in the SG cattle group was higher than that of the UG group. Notably, the results for correlation analyses showed that the *Paludibacter* had a positive effect on these three potential pathogenic bacteria. Moreover, we found that excessive intake of sodium and iron is associated with these microbes that have potential pathogenic risks. Our results indicate that the continued global saline-alkaline degradation of grasslands will significantly elevate the risk of animal diseases, and may also pose a serious threat to human health through environmentally mediated transmission.

## Simple Summary

The serious environmental problems caused by grassland saline-alkaline degradation have been widely recognized, yet we still lack knowledge on whether grassland saline-alkaline degradation further increases the environmental transmission risk of pathogens from grazing animals. This study examined the impact of grassland saline-alkaline degradation on the potential fecal pathogens in cattle. Our results provide new experimental evidence that grassland saline-alkaline degradation-induced excessive iron and sodium intake are mainly responsible for the elevation of potential fecal pathogenic bacteria in cattle. These findings suggest that global grassland saline-alkaline degradation potentially increases animal disease risk, thereby posing a serious threat to human health via environmental transmission.

## 1. Introduction

Approximately 2.7 million people are estimated to die globally each year from zoonoses, which are caused by the environmental transmission (water, air, soil, etc.) of pathogens mainly found in the excreta of domesticated animals [1]. Notably, ruminants are the primary producer of zoonotic strains such as *Escherichia coli* (*E. coli*), *Salmonella*, *Campylobacter jejuni*, and *Listeria monocytogenes* among all types of domesticated animals, and thus pose a significant threat to human health [1,2]. In recent decades, extensive research has been conducted to assess pathogen risk in stall-fed ruminants, with particular attention to the effects of dietary changes [3,4,5], temperature fluctuations [6,7], ambient humidity [6,8], and air quality [7,9,10]. Conversely, research on pathogen risk assessment in grazing systems remains limited, with scattered studies focusing on the effects of seasonal variations [11] and animal behavior [12] on animal pathogens. Actually, the most critical issue facing the current grazing system is that global grasslands are experiencing degradation. Indeed, approximately half of the global grasslands have experienced degradation to some extent owing to climate change and anthropogenic activities in the past few decades [13]. In particular, grassland saline-alkaline degradation is widespread among various types of grassland degradation in arid and semi-arid regions globally [14]. This phenomenon is primarily driven by human-induced overgrazing, which increases soil porosity and enhances capillary action, thereby facilitating the upward movement of saline groundwater, and ultimately leading to the accumulation of salts in the soil surface through evapotranspiration [15,16]. This saline-alkaline degradation is characterized primarily by the decline of perennial plant species such as *Leymus chinensis* and the proliferation of annual species like *Suaeda glauca* [17]. At present, extensive studies have demonstrated that grassland saline-alkaline degradation poses significant environmental threats. For instance, the saline-alkaline degradation of grassland has significantly reduced plant coverage, thereby impairing the vegetation’s ability to stabilize soil and mitigate wind erosion [17,18]. Previous studies also further found that grassland saline-alkaline degradation reduced the carbon sequestration capacity of both plants and soils, thereby resulting in increased carbon loss and decreased ecosystem stability [16,19,20]. In addition, the degree of water resource pollution has also been increased in the grassland saline-alkali degradation area [21]. Obviously, current research on environmental threats related to grassland salinization and degradation primarily focuses on vegetation dynamics and soil physicochemical property alterations, but generally overlooks grazing animals as key vectors for pathogen transmission [15,22]. The current lack of understanding regarding how grassland saline-alkaline degradation influences the transmission risk of pathogens in grazing livestock represents a critical knowledge gap, particularly given its potential dual threat to both animal and human health.

Despite the lack of the above understanding, previous studies on relevant topics have still provided us with important inspiration. Previously published literature has demonstrated that a change in food resources is a key factor influencing the release of animal pathogens [23,24]. Grassland saline-alkaline degradation undoubtedly represents a significant alteration in food resources for grazing animals. Indeed, previous studies have demonstrated that the fiber content of grassland plant resources declines linearly with increasing grassland degradation, attributable to a reduction in perennial species and a corresponding increase in annual species [25,26]. A reduction in fiber intake may increase the relative abundance of fecal pathogenic bacteria in grazing ruminants by inhibiting the production of short-chain fatty acids [27]. Moreover, research has also demonstrated that grassland saline-alkaline degradation can increase the fat content of plant resources [28]. Consequently, the concentration of long-chain fatty acids in plants from saline-alkaline degraded grasslands may be elevated, potentially contributing to a reduction in the relative abundance of fecal pathogenic bacteria in grazing animals by inhibiting key transcriptional activators of these pathogens [29]. Moreover, the most significant indicator of grassland saline-alkaline degradation is increased ion content in both soils and plants [30,31], which may result in the excessive ion intake of grazing ruminants. However, it still remains unknown whether alterations in ion intake would further cause changes in fecal pathogens in ruminants. In addition, the potential changes in soil and plant pathogenic bacteria induced by grassland degradation may increase the risk of ingestion of pathogenic bacteria by livestock, but it may be difficult for these pathogenic microbes to colonize the animal gut due to their weaker competitiveness relative to the native microbes [32]. Based on the above, we hypothesized that grassland saline-alkaline degradation may further increase the number of pathogenic bacteria in the excrement of grazing ruminants, thereby threatening the environment and human health.

Accordingly, this study was conducted to assess the impact of grassland saline-alkaline degradation on fecal pathogenic bacteria in grazing ruminant cattle. In addition, this study also conducted a preliminary investigation into the underlying mechanisms by analyzing changes in nutrient intake associated with saline-alkaline grassland degradation. The important significance of our research is that it integrates the environmental impacts of grazing livestock into grassland ecosystem assessment systems, thereby providing new insights into the ecological consequences of grassland saline-alkaline degradation. On the other hand, our work not only fills a critical knowledge gap in the “plant-animal” continuum of saline-alkaline degraded grasslands but also establishes a scientific foundation for developing sustainable grazing management strategies. These strategies can mitigate environmental risks while contributing to public health security.

## 2. Materials and Methods

The study sites are located in the Songnen grasslands in western Jilin Province, northeast China (44°45′ N, 123°45′ E), where most of the grasslands have undergone severe saline-alkaline degradation owing to overgrazing, mostly for cattle and sheep. The dominant steppe species in the Songnen grasslands is the perennial *Leymus chinensis*, and other common species are *Phragmites australis*, *Kalimeris indica*, *Calamagrostis epigejos*, *Artemisia mongolica*, etc. After saline-alkaline degradation, the dominant steppe species is replaced by *Chloris virgata*, and other common species are replaced by *Artemisia anethifolia*, *Kochia sieversian*, *Suaeda glauca*, etc. In this area, we selected two representative grassland types (approximately 600 hectares per grassland and 10 km apart) for grazing experiments, one of which was undegraded and the other of which was severely saline-alkaline degraded. The classification of grassland saline-alkaline degradation levels is determined according to ECe (Electrical Conductivity of the Soil’s Saturated Extract), pH and FVC (Fractional Vegetation Coverage). Specifically, when the soil ECe > 16 dS m^−1^, a pH value above 7 and grassland FVC of less than 40% is regarded as a saline-alkaline degraded grassland [17,33,34]. In this study, the saline-alkaline degradation of grassland areas primarily manifested as increased levels of sodium and iron ions in soil. All experimental procedures involving animals were carried out according to the principles and responsibilities outlined in the Northeast Normal University’s (Changchun, China) guidelines for animal research (Approval Number: 10200-2023-03).

### 2.1. Design of Experiment 1

**Figure 1:**
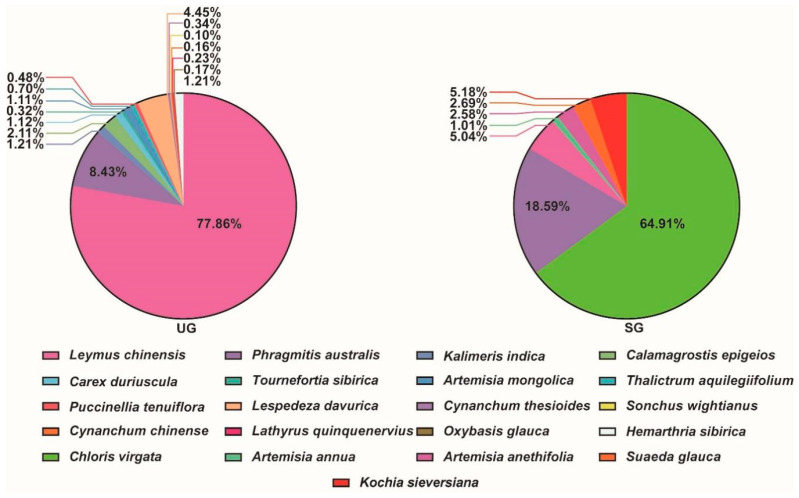
The plant composition and proportion of undegraded (UG) and severe saline-alkaline degraded grasslands (SG).

We selected two grassland areas (approximately 600 hectares per area) from the Changling County Xingmu Farming Co-operative Society (44°28′ N, 123°47′ E) for grazing experiments, one of which was undegraded and the other of which had already experienced severely saline-alkaline degradation. Approximately 400 Simmental bulls were grazed in each area from 1 June to 30 September 2023. We selected twenty bulls with similar body weight (408.05 ± 9.35 kg) and age (360.35 ± 10.47 d), which were divided into two treatment groups based on their grazing areas: (1) grazing on undegraded grassland (UG), and (2) grazing on severely saline-alkaline degraded grassland (SG), with 10 cattle in each group. Fences and shade sheds were installed in each area to ensure adequate resting areas and access to water for the cattle. The stocking rate in this experiment was maintained at approximately 0.23 cattle units per hectare to support sustainable grazing practices. The plant composition of saline-alkaline degraded grassland and undegraded grassland is presented in Figure 1. Throughout the grazing feeding period, all the cattle had free access to fresh water but without concentrate supplementation.

### 2.2. Design of Experiment 2

Given the significant variation in plant composition across different sites within the grazing land, such spatial heterogeneity may introduce considerable variability in the foraging behavior of grazing animals, thereby potentially compromising the reliability of Experiment 1’s results. To validate the reliability of the results from Experiment 1, a foraging behavioral experiment from our team was embedded within the current study. A two-factor design (grassland type: undegraded grassland vs. severely saline-alkaline degraded grassland) with three independent replicates per treatment was used in this experiment. Three representative 100 m × 100 m plots were selected for each grassland type, with spatial separation maintained between plots (spacing > 1 km) to ensure independence. Each plot was enclosed by fencing to prevent the animals’ movement between plots. Eighteen male Simmental bulls with similar weight (405.10 ± 1.33 kg) and age (359.67 ± 2.43) used in this experiment were selected from the same Co-operative Society as in Experiment 1. Three cattle were randomly selected and grazed freely within each plot for 7 consecutive days at the end of June, July, August, and September 2024. The stocking rate in this experiment was similar to that of Experiment 1, which was maintained at approximately 0.23 cattle units per hectare. In addition, cattle had free access to fresh water but without concentrate supplementation throughout the grazing period.

### 2.3. Monitoring of Plants Consumed by Cattle

Four Simmental cattle were randomly selected from each group in Experiment 1 for foraging monitoring. Moreover, all the Simmental cattle from Experiment 2 were used for foraging monitoring during the last three days of each month. As described by previous research, the action camera (GoPro Hero4; Resolution: 1920 × 1080; Ruhong Co., Ltd., Shenzhen, China) was hung from the lower part of the cattle’s neck using a strap to provide a field of view aimed downwards towards the vegetation being consumed, thereby clearly videoing the bulls foraging during the daily grazing period. We accurately recorded the plant species foraged and their corresponding numbers of bites because of the high video resolution, which could be viewed at slower speeds or replayed. Throughout each experimental period, we recorded the grazing behavior of cattle for 60 min each day because a 60 min duration was sufficient to obtain the foraging behavior of grazing animals [35,36]. We determined the plants and quantities consumed by grazing cattle based on the average of the observations made by three observers [35,37,38]. If the recording errors of the three observers were within 5%, we considered the recording results reliable; otherwise, we would replace the observers and re-record. Then, the weight of one bite of each plant foraged by the cattle was obtained via calculating the average of the total weight of 100 bites of each plant, which was simulated to be harvested artificially. Artificial harvesting simulations were conducted in accordance with the video imagery and the plants were weighed using a weighing scale. Thereafter, the total amount of each plant consumed by the cattle daily was calculated for further determination of daily dry matter intake (DMI). In addition, the diversities (simpson index; shannon index; peilou’s evenness) of plants foraged by cattle were calculated according the number of species and the abundance of plants; the functional indices (functional evenness, FEve; functional dispersion index, FDis; functional divergence index, Fdiv; Rao’s quadratic entropy index, RaoQ) of plants foraged by cattle were calculated according to the ether-extract (EE), crude protein (CP), neutral detergent fiber (NDF), acid detergent fiber (ADF) and mineral element content in this study. Functional indices were calculated using R Statistical Software (v4.1.2; R Core Team 2021). The module [39] is presented in the Supplementary Materials. The calculation formula for plant diversity was as follows: (1) Simpson index: D = 1 − ∑(*Ni*/*N*)^2^, where *Ni* represents the number of individuals of the *i*-th species, and *N* is the total number of individuals; (2) Shannon index: H = −∑(*Pi*)(ln*Pi*), where *Pi* represents the proportion of individuals of this species to the total number of individuals; (3) Peilou’s evenness: J = −∑(*Pi* ln *Pi*)/ln *S*, where *Pi* represents the proportion of the total number of individuals of the *i*-th species to the total number of individuals; *S* represents the total number of species.

### 2.4. Sample Collection

In Experiment 1 and Experiment 2, the collection of plants was based on the foraging behavior of grazing cattle. The plants from Experiment 1 were collected weekly, and the plants from Experiment 2 were collected during the final week of each month throughout the experimental period. Eventually, we collected 12 types of plants (*Leymus chinensis*, *Phragmites australis*, *Kalimeris indica*, *Calamagrostis epigeios*, *Carex duriuscula*, *Tournefortia sibirica*, *Lespedeza davurica*, *Artemisia mongolica*, *Thalictrum aquilegiifolium*, *Chloris virgata*, *Artemisia annua*, and *Suaeda glauca*) in Experiment 1, and 14 types of plants (*Chloris virgata*, *Kochia scoparia*, *Leymus chinensis*, *Phragmites australis*, *Kalimeris indica*, *Calamagrostis epigeios*, *Carex duriuscula*, *Tournefortia sibirica*, *Chloris virgata*, *Artemisia scoparia*, *Artemisia mongolica*, *Knorringia sibirica*, *Lespedeza davurica*, and *Lespedeza chinensis*) in Experiment 2. Then, the plant samples collected weekly from Experiment 1 and Experiment 2 were thoroughly mixed according to the cattle’s foraging behavior to obtain the daily ration for each cattle. For each of the plants, 1.2 to 1.5 kg were collected. Subsequently, the plant samples were dried at 65 °C for 72 h, milled to pass through a 1 mm mesh screen, and then stored in sealed bags (150 mm × 220 mm) at 4 °C until their chemical composition was analyzed. In addition, approximately 10 g spot fecal samples of each bull from Experiment 1 were collected via the rectal extraction method on the final day of the experiment at 0600 and 1800 h. The samples collected from each cattle were composited and immediately stored in liquid nitrogen until analysis of the fecal bacterial community was conducted. The fecal samples collected at 0600 and 1800 were mixed in equal proportions before conducting the measurement.

### 2.5. Analysis of the Nutritional Composition of Plants

Samples of each plant were sent to the Animal Nutrition Laboratory of Northeast Agricultural University (Harbin, China) for nutrient analysis using wet chemistry methods. The contents of the dry matter (DM, method 934.01), ash (method 942.05), EE (method 920.39), and CP (method 988.05) in the plants were assayed in accordance with the procedures of AOAC International (2002) [40]. The NDF and ADF contents were measured based on the method described by a previous study [41], in which the heat-stable α-amylase was used to treat the plants. The mineral element concentrations were measured using an inductively coupled plasma-optical emission spectrometer (ICP-6800S, Meixi Instrument Co., Ltd., Shanghai, China). For detailed methodological procedures, please refer to the study conducted by Staniek and Wójciak (2018) [42].

### 2.6. Analysis of the Bacterial Community Composition in Feces

Total DNA in the feces was extracted using a DNA extraction kit (Shengye Biotech Co., Ltd., Shanghai, China), following the manufacturer’s instructions, and was used as the template for 16S rDNA sequencing analysis. To ensure the integrity and suitability of the extracted DNA, both concentration and purity were evaluated using a Thermo NanoDrop One spectrophotometer (Shengye Biotech Co., Ltd., Shanghai, China). The V3–V4 hypervariable regions of the bacterial 16S rDNA gene were amplified via PCR using the specific primer pair 338F (5′-ACTCCTACGGGAGGCAGCA-3′) and 806R (5′-GGACTACHVGGGTWTCTAAT-3′). For accurate sample discrimination, each primer pair was tagged with a unique eight-base pair barcode sequence. The PCR reaction mixture consisted of 1 μL of forward primer, 1 μL of reverse primer, 25 μL of 2× Premix Taq (Shengye Biotech Co., Ltd., Shanghai, China), 50 ng of genomic DNA, and nuclease-free water to a final volume of 50 μL. Amplification was performed under the following thermal cycling conditions: initial denaturation at 94 °C for 5 min, followed by 30 cycles of 94 °C for 30 s, 52 °C for 30 s, and 72 °C for 30 s, with a final extension at 72 °C for 10 min. The resulting PCR amplicons were resolved by electrophoresis on a 1% (*w*/*v*) agarose gel [43]. The PCR products were purified using an OMEGA DNA purification column (Shengye Biotech Co., Ltd., Shanghai, China). The purified products were quantified using a Quant-iT PicoGreen dsDNA Assay Kit (Gene Company Limited) in accordance with the kit’s instructions. The DNA extraction and PCR process for all samples were repeated three times to ensure the reliability of the results. The number of gene reads for each sample is approximately 40,000. The DNA fragments were paired-end sequenced by an Illumina miseq/novaseq. UCHIME (v 4.2) [44] was used for de novo chimera checking, and USEARCH (v 5.2.32) [45] was used for operational taxonomic unit (OTU) identification in order to identify similar sequences that had >97% similarity. All samples were standardized using the DESeq2 method [46]. In fact, ASV-based methods (such as DADA2) offer advantages in resolution and reproducibility. The major reason for adopting the OTU method based on 97% similarity in this study was to maintain direct comparability with the extensive historical research data available within this field. To address these issues as thoroughly as possible, we implemented rigorous quality control and chimera removal procedures. We are confident that the core differences observed at the genus and family levels within the community are reliable, which is the primary focus of this study. However, future work employing ASV methods for strain-level analysis may prove more meaningful. Given that the Greengenes 16S rRNA gene database is now relatively outdated and reliance on a single database may compromise taxonomic accuracy, representative sequences for bacterial OTUs were assigned to both the Greengenes 16S rRNA gene database (version gg_13_8) [47] and the Silva database [48]. We used the Greengenes database and the Silva database to better screen out potential pathogenic microorganisms. Raw reads of the 16S rDNA gene sequencing of feces microbiota are available at NCBI (PRJNA1331944).

### 2.7. Statistical Analysis

Firstly, all data used in this study met the assumptions of normality and homogeneity of variance, as confirmed by the Shapiro–Wilk test and Levene’s test, respectively, and the data that passed the tests were subsequently analyzed. R Statistical Software (v4.1.2; R Core Team 2021) was used to analyze all of the experiment’s data. No missing or outlier data were observed in this study. In the event of such occurrences, these values would be excluded by default according to our predefined data handling protocol. ANOVA was used to analyze the effects of grassland saline-alkaline degradation on the nutrient intake (data from Experiment 1 and Experiment 2) and fecal bacterial communities (n = 10/group) of cattle. In addition, the ANOVA was also used to examine differences in nutrient intake among cattle (*n* = 3/plot) at three plots within a grassland type (undegraded grassland or severely saline-alkaline degraded grassland). To analyze the effects of nutrient intake on fecal bacterial relative abundance, we used linear mixed effects model [49]. Further, we also determined the relative importance of various mineral elements and foraging plant species diversity for fecal potential pathogenic bacteria using linear mixed effects models. In these models, nutrient intakes and foraging plant species diversity were taken as fixed factors and grassland types (undegraded grassland and severely saline-alkaline degraded grassland) were taken as random factors. Detailed information about the model can be found in the Supplementary Materials. After testing, the model conforms to independence and homoscedasticity and the residuals in the model also conform to normality (Shapiro–Wilk test). Significant differences were declared at *p* ≤ 0.05, and marginally significant differences were defined at 0.05 < *p* ≤ 0.10.

## 3. Results

### 3.1. Variation in Fecal Bacterial Community

**Figure 2:**
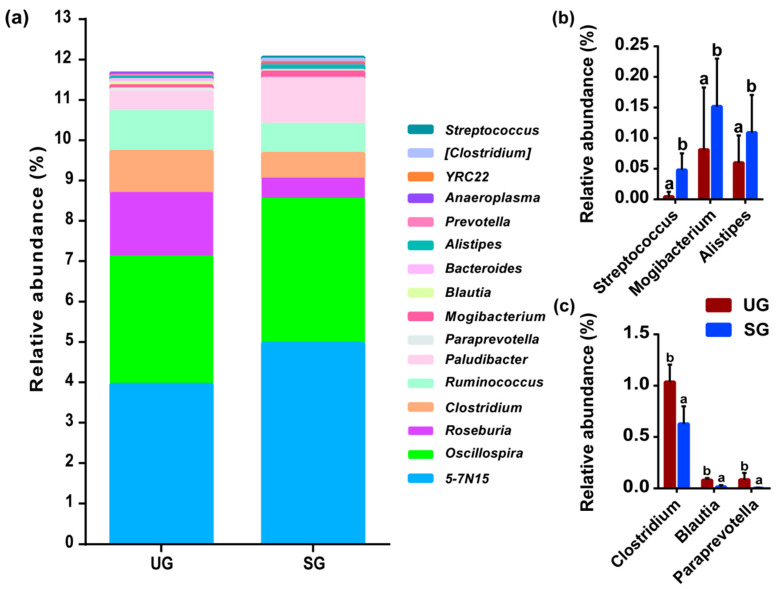
Grassland saline-alkaline degradation increased the abundance of potential pathogenic bacteria but reduced the beneficial bacteria abundance in the feces of grazing cattle. (**a**) The 16 most abundant bacterial genera in feces. (**b**) Genera containing pathogenic bacteria. (**c**) Genera containing bacteria associated with immune modulation; bars represent standard deviation; values with different letters indicate significant differences (*p* < 0.05). UG—undegraded grassland, SG—severe saline-alkaline degraded grassland.

The relative abundances of the 16 most abundant bacteria genera are given in Figure 2a. The most abundant bacteria genus was 5-7N15, followed by *Oscillospira* and *Roseburia*, etc. Among these genera, the relative abundances of potential pathogenic bacteria including *Streptococcus*, *Mogibacterium*, and *Alistipes* in the SG group were higher than those of the UG group (Figure 2b; *p* < 0.05). Conversely, cattle grazing on severe saline-alkaline degraded grassland significantly decreased the relative abundance of fecal bacteria associated with immune modulation that include *Clostridiu*, *Blautia*, and *Paraprevotella* compared with the cattle grazing on undegraded grassland (Figure 2c; *p* < 0.05).

### 3.2. Correlation Among Various Bacteria

**Figure 3:**
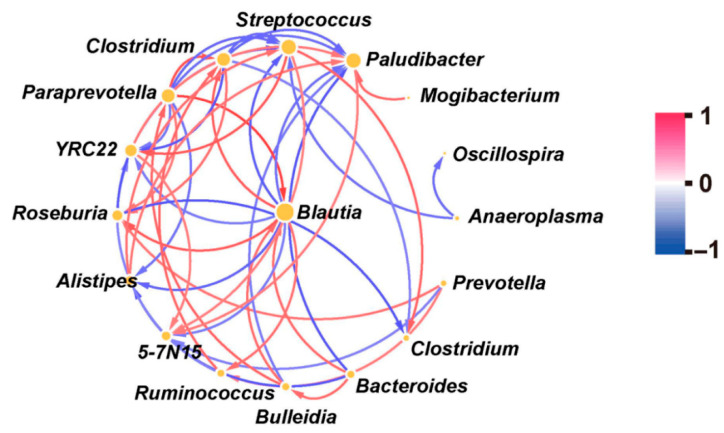
Analysis of the correlation among various fecal bacteria genera.

Figure 3 showed that the *Paludibacter* plays the most important role among these bacteria, as it not only had a positive effect on the potential pathogenic bacteria *Streptococcus*, *Mogibacterium*, and *Alistipes*, but also had a negative effect on *Clostridium*, *Blautia*, and *Paraprevotella* (*p* < 0.05). In addition, *Streptococcus* had a negative effect on *Clostridium*, *Blautia*, and *Paraprevotella* (*p* < 0.05); *Alistipes* also had a negative effect on *Blautia* and *Paraprevotella* (*p* < 0.05). However, *Mogibacterium* had no significant effect on the other bacteria (*p* > 0.10).

### 3.3. Foraging Diversity

**Figure 4:**
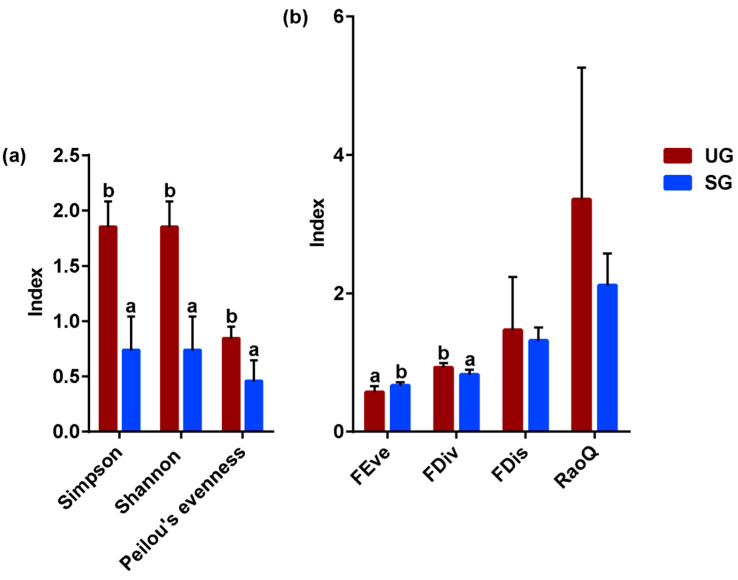
Grassland saline-alkaline degradation reduced diversities of plant species (**a**) and functions (**b**) foraged by cattle. FEve—functional evenness, FDiv—functional divergence index, FDis—functional dispersion, RaoQ—Rao’s quadratic entropy index; bars represent standard deviation; values with different letters indicate significant differences (*p* < 0.05). UG—undegraded grassland, SG—severe saline-alkaline-degraded grassland.

We observed that the plant species diversity (Simpson index, the Shannon index, and Peilou’s evenness) foraged by cattle grazing on undegraded grassland was significantly higher than that foraged by cattle grazing on severely saline-alkaline degraded grassland (Figure 4a; *p* < 0.05). Regarding the plants’ functional diversity, we found that the FEve index in the SG group was higher (which means a more balanced intake of nutrients) than that of the UG group, whereas the FDiv index was lower (which means a lower nutritional function) (Figure 4b; *p* < 0.05). Moreover, no difference for RaoQ and FDis was observed between the two groups (*p* > 0.10).

### 3.4. Proximate Nutrient Intake

**Figure 5:**
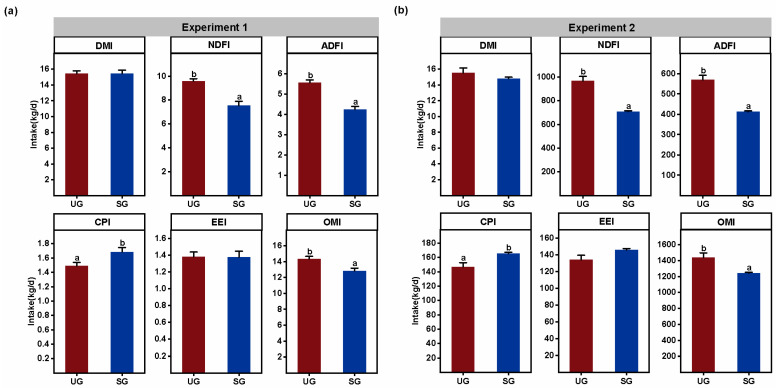
Grassland saline-alkaline degradation altered the nutrient intake of grazing cattle in Experiment 1 (**a**) and Experiment 2 (**b**). DMI—dry matter intake, NDFI—neutral detergent fiber intake, ADFI—acid detergent fiber intake, CPI—crude protein intake, EEI—ethyl ether extract intake, OMI—organic matter intake; bars represent standard deviation; values with different letters indicate significant differences (*p* < 0.05). UG—Undegraded grassland, SG—severe saline-alkaline-degraded grassland.

In both Experiment 1 and Experiment 2, cattle grazing on severely saline-alkaline degraded grassland showed not only an increase in CPI, but also a decrease in NDFI, ADFI and OMI compared with the cattle grazing on undegraded grassland (Figure 5; *p* < 0.05). In addition, the DMI and EEI were not altered between the two treatment groups (*p* > 0.10). Further, we also observed from the results of Experiment 2 that the cattle showed no difference in foraging plant composition and proximate nutrient intake among three plots within the same grassland type (Table 1; *p* > 0.10), which indicated that different spatial areas within the same grassland type do not alter the foraging plant composition and proximate nutrient intake of cattle.

### 3.5. Mineral Element Intake

**Figure 6:**
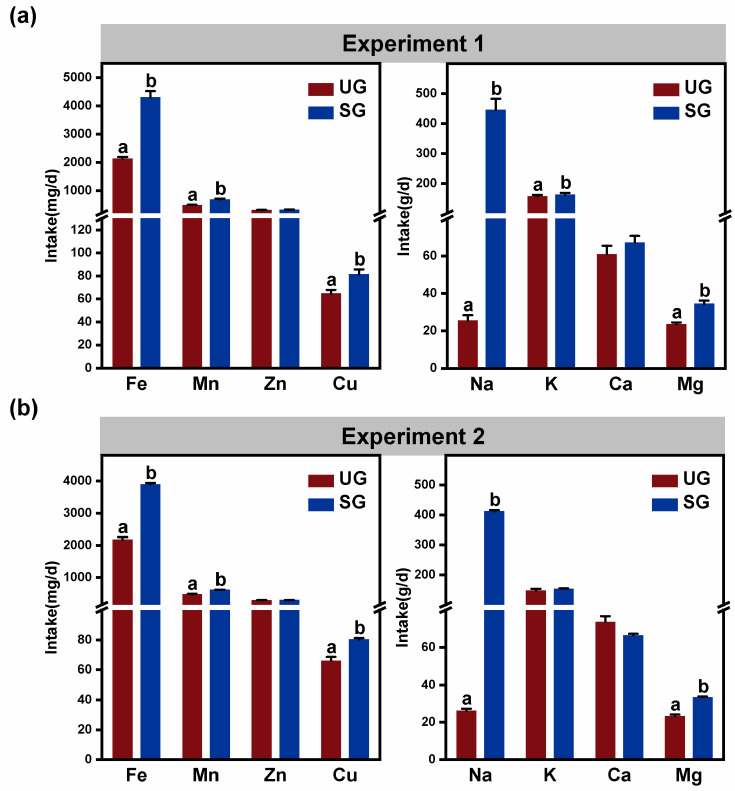
Grassland saline-alkaline degradation increased mineral elements intake of grazing cattle in Experiment 1 (**a**) and Experiment 2 (**b**). Bars represent standard deviation; values with different letters indicate significant differences (*p* < 0.05). UG—undegraded grassland, SG—severe saline-alkaline degraded grassland.

In both Experiment 1 and Experiment 2, the results for mineral element intake showed that cattle in the SG group had higher Iron (Fe), Manganese (Mn), Copper (Cu), Sodium (Na), Potassium (K) and Magnesium (Mg) intake than that of the UG group (Figure 6; *p* < 0.05). However, there was no significant difference for Zinc (Zn) and Calcium (Ca) intake between the two groups (Figure 6; *p* > 0.10). Additionally, we also observed from the results of Experiment 2 that cattle showed no difference in foraging plant composition and mineral element intake among three plots within the same grassland type (Tables S1 and S2; *p* > 0.10), which indicated that different spatial areas within the same grassland type do not alter the foraging plant composition and mineral element intake of cattle.

### 3.6. The Relative Importance of Various Factors for the Key Fecal Bacteria

We first analyzed the effects of various factors on key fecal bacteria, and found that the intake of Fe, Na, Cu, Zn, Mn, and Mg and the Simpson index of foraging plants might be associated with key fecal bacteria (*p* < 0.05; Table S3). Further, we also analyzed the relative importance of various mineral elements and foraging plant species diversity for the key fecal bacteria (Table 1). We observed that the intake of Fe relative to other factors was more strongly associated with the improvement in the relative abundance of fecal *Streptococcus* and *Paludibacter*, while the intake of Na relative to other factors was more closely linked to the increase in the relative abundance of fecal *Alistipes*.

## 4. Discussion

Our results from Experiment 1 found that grassland saline-alkaline degradation increased the relative abundance of fecal potential pathogenic bacteria in cattle by altering their nutrient intake. However, the design of Experiment 1 suffered from insufficient true replication, with each grassland type represented by only a single plot, which may compromise the reliability of the study’s conclusions. To address this limitation, the supplemental Experiment 2 was embedded within the present study. We found that cattle showed no difference in foraging plant composition and nutrient intake among three plots within the same grassland type (Tables S1 and S2). Moreover, the results regarding the differences in nutrient intake of cattle between the two grassland types were similar in Experiment 1 and Experiment 2. Therefore, to address the limited reproducibility in Experiment 1, we conducted Experiment 2 as a supplementary validation to indirectly strengthen the credibility of the findings from Experiment 1 concerning the impact of grassland saline-alkaline degradation on cattle fecal potential pathogen abundance. Nevertheless, Experiment 2 currently lacks replication across time and space, which should be addressed in future studies to further confirm the robustness of these results. Collectively, our research provides the first empirical data that grassland saline-alkaline degradation might increase the relative abundance of fecal potential pathogenic bacteria in cattle. Such a result indicates that cattle grazing on severely saline-alkaline degraded grassland might potentially contribute to an increase in the transmission risk of pathogenic bacteria, thereby threatening the health of animals and even humans.

Specifically, our results showed that cattle grazing on severely saline-alkaline degraded grassland might significantly increase the relative abundance of fecal *Streptococcus*, *Mogibacterium*, and *Alistipes* (Figure 2b). Previous studies have demonstrated that these three bacterial genera belonging to potential pathogenic bacteria had the potential to increase disease-associated dysbiosis [50,51,52]. More importantly, fecal *Streptococcus* is suggested as an opportunistic pathogen and also a suitable indicator of the survival rates of pathogens due to their enhanced survival duration and increased resistance to thermal stress [2,53]. Previous studies have indicated that *Streptococcus* is transmitted through direct contact with respiratory secretions or indirect contact with contaminated surfaces, indicating that the interindividual transmission of this bacterium occurs readily under conducive conditions [54,55]. On the other hand, *Alistipes* exhibits broad transmission potential, with frequent interindividual spread, and can even be transmitted between mothers and infants [56,57,58]. Thus, the increase in fecal potential pathogenic bacteria abundance would potentially increase the risk of animal as well as human diseases through environmentally mediated transmission. In addition, we found that cattle grazing on severely saline-alkaline degraded grassland might significantly reduce the relative abundance of fecal bacteria associated with immune modulation, including *Clostridium*, *Blautia*, and *Paraprevotella* (Figure 2c) [59,60,61], indicating that an increase in potential fecal pathogenic bacteria may affect animal health by decreasing the abundance of bacteria associated with immune modulation. Indeed, further correlation analyses among various bacteria genera showed that these potential pathogenic bacteria had a negative effect on *Clostridiu*, *Blautia*, and *Paraprevotella* (Figure 3). More notably, the results of the correlation analysis indicated that the relative abundance of *Paludibacter* in the feces of cattle was positively correlated with the relative abundance of potential pathogenic bacteria. However, the underlying mechanism of the association between *Paludibacter* and potential pathogenic bacteria remains unclear and warrants further investigation. Therefore, our results suggested that grazing cattle consuming plants from severely saline-alkaline degraded grasslands may lead to an increased abundance of potential pathogenic bacteria in their feces, while decreasing the abundance of certain beneficial bacteria.

Experimental evidence has shown that fecal bacterial composition is primarily influenced by dietary nutrient characteristics [62,63,64]. Thus, the changes in plant resources resulting from grassland saline-alkaline degradation in this study may serve as a critical driver behind the observed shifts in fecal bacterial communities in cattle. Indeed, a previous study has demonstrated that sheep grazing on saline-alkaline degraded grassland improved their EE intake [65], which has the ability to reduce the relative abundance of fecal pathogenic bacteria of animals [66]. However, our results found that the EE intake did not differ between the two groups (Figure 5), which may be attributed to cattle having lower foraging selectivity [35], resulting in similar dietary fat content when grazing on both saline-alkaline degraded and undegraded grasslands. In addition, we found that cattle grazing on severely saline-alkaline degraded grasslands exhibited reduced intake of NDF and ADF (Figure 5). Although reductions in NDF and ADF intake are often associated with increased relative abundance of fecal pathogenic bacteria in ruminants [27], the results of the linear mixed effects modeling analysis in this study revealed no significant association between fiber intake and fecal potential pathogen abundance. This phenomenon might be attributed to the relatively smaller variation in fiber intake between groups compared to that observed in previous studies [67,68]. Therefore, we highlighted that changes in proximate nutrient intake have no obvious correlation with the increased abundance of fecal potential pathogenic bacteria.

**Figure 7:**
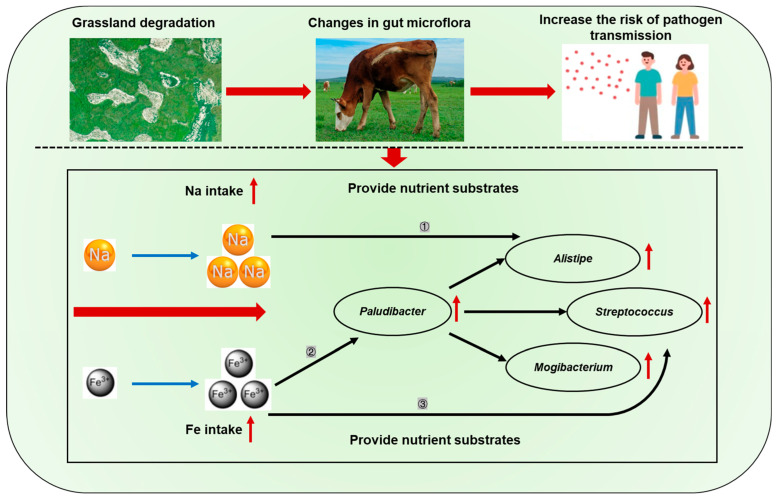
The underlying mechanisms of grassland saline-alkaline degradation affecting the abundance of fecal potential pathogenic bacteria in cattle. ① ② ③ represent the three pathways of influence of grassland saline-alkaline degradation on fecal potential pathogenic bacteria; black arrows represent positive regulatory effects; red arrows represent that the related parameters of cattle grazing on saline-alkaline degraded grassland were higher (*p* < 0.05) than those of cattle grazing on healthy grassland.

Apart from proximate nutrient intake, our results also showed that plant species diversity foraged by cattle grazing on undegraded grassland was significantly higher than that foraged by cattle grazing on severely saline-alkaline degraded grassland (Figure 4a). High foraging diversity typically indicates that livestock have access to a broader range of nutrients, which may promote intestinal health and consequently reduce the abundance of fecal pathogenic bacteria. This conjecture was proven by the results of the linear mixed effects modeling analysis in this study, which found that there is a significant negative correlation between plant species diversity foraged by cattle and the relative abundance of *Streptococcus*, *Paludibacter*, and *Alistipes* (Table S3). In addition, we also found that cattle grazing on severely saline-alkaline degraded grasslands showed an increase in Fe, Mn, Cu, Na, K, and Mg intake compared with the cattle grazing on undegraded grassland (Figure 6). Particularly for Fe and Na intake, the cattle grazing on severely saline-alkaline degraded pastures exhibited Fe intake levels significantly exceeding their daily dietary requirements when compared to the nutritional requirements proposed by the National Research Council (NRC 2001). Thus, the excessive intake of Fe and Na ions may be associated with the relative abundance of potential pathogenic bacteria in cattle feces. Further, we determined the relative importance of various mineral elements and foraging plant species diversity for fecal potential pathogenic bacteria using linear mixed effects models. We observed a strong correlation between iron intake and the increase in the relative abundance of fecal *Streptococcus* (Table 1). Similarly, previous studies have demonstrated that excessive Fe intake results in unabsorbed Fe passing into the colon, where it can be utilized by potentially pathogenic bacteria to support their growth [69,70,71,72]. Further study has also demonstrated that sheep consuming excessive amounts of Fe showed a linear increase in the relative abundance of *Proteobacteria* [73], which consisted mainly of pathogenic bacterial members (Shin et al., 2015; Petri et al., 2013) [23,74]. In addition, research demonstrates that iron is an essential nutrient required for the growth and proliferation of *Streptococcus*, which can exploit iron derived from hemoglobin to enhance its ability to colonize host tissues and grow on host proteins [75,76,77]. This capacity for Fe utilization is closely linked to the pathogen’s survival, environmental adaptation, and virulence. Meanwhile, the Fe intake is also strongly associated with the increase in the relative abundance of fecal *Paludibacter* (Table 1). Hence, the increased Fe intake may contribute to the enhancement of fecal potential pathogenic bacteria abundance by providing a favorable ecological niche and through indirect modulation of fecal *Paludibacter* abundance. Moreover, we also observed from the results of the linear mixed effects models that Na intake has the strongest correlation with the increase in the relative abundance of fecal *Alistipes* (Table 1). This observation is in line with the study from Hamad et al. (2022) [78], in which high dietary Na intake has been shown to improve the relative abundance of gut *Alistipes* in mice because of an underrepresentation of lactic-acid-producing bacteria. Our results suggest that changes in Fe and Na intake induced by grassland saline-alkaline degradation were two main factors influencing the relative abundance of fecal potential pathogenic bacteria in cattle (Figure 7), but the impact mechanisms still need to be further explored.

## 5. Conclusions

Cattle grazing on severely saline-alkaline degraded grasslands showed an increase in Fe and Na intake. The increased Fe intake relative to other factors might contribute most to the enhancement of the relative abundance of fecal *Streptococcus* belonging to potential pathogenic bacteria by providing an advantageous niche for them as well as indirect regulation of the abundance of *Paludibacter*, which had a positive effect on the potential pathogenic bacteria. In addition, the increased Na intake relative to other factors might also contribute most to the enhancement of the relative abundance of fecal *Alistipes*. In general, our study provides experimental evidence that excessive Fe and Na ions in the plants of saline-alkaline grasslands may potentially represent one of the contributing risk factors associated with elevated pathogen emissions in livestock feces, thereby further exacerbating the adverse environmental effects of grassland degradation. However, although the findings from Experiments 1 and 2 suggest that the conclusions of this study are reasonably reliable, more large-scale animal studies under more rigorous experimental designs are still required in the future. Furthermore, given that the current study is limited to cattle, it would be valuable to examine whether other grazing species exhibit distinct responses to such alterations of fecal potential pathogens. Overall, our study offers a novel perspective on the assessment of environmental risks associated with grassland degradation and provides a robust theoretical foundation for sustainable grazing management practices. In future grazing management practices, we recommend that livestock in this region be routinely vaccinated against potential pathogens. Concurrently, manure deposited on the grassland should be systematically treated through methods such as composting and the application of lime or aqueous ammonia to reduce pathogen load and mitigate environmental risks. In addition, we also recommend revisiting and extending this research at critical time intervals corresponding to the mid-21st century (2051–2075) and late 21st century (2076–2100) based on climate prediction models constructed by previous researchers, particularly in relation to projected changes in extreme rainfall events.

## Figures and Tables

**Table 1:** Summary of linear mixed models analyzing the relative importance of various mineral elements and foraging plant species diversity for key fecal bacteria. Mineral element intake and foraging plant Simpson index were taken as fixed factors; grassland types (undegraded grassland and severe saline-alkaline degraded grassland) were taken as random factors.

Key Bacteria	Variable	DF	F-Value	*p*-Value
*Paludibacter*	Fe	4	196.67	<0.01
	Cu	4	0.50	0.52
	Fe	4	249.54	0.05
	Mg	4	1.97	0.23
	Fe	4	260.34	<0.01
	Simpson index	4	2.28	0.21
*Streptococcus*	Fe	4	51.08	<0.01
	Cu	4	1.04	0.37
	Fe	4	54.1	<0.01
	Mg	4	1.53	0.28
	Fe	4	38.12	<0.01
	Simpson index	4	0.04	0.84
*Alistipes*	Na	4	177.87	<0.01
	Cu	4	9.19	0.11
	Na	4	115.61	<0.01
	Mg	4	4.51	0.10
	Na	4	61.85	0.02
	Simpson index	4	0.49	0.52

## Data Availability

Raw reads of the 16S rDNA gene sequencing of feces microbiota are available at NCBI (PRJNA1331944). Other data are available from the corresponding author upon reasonable request.

## Supplementary Materials

The following supporting information can be downloaded at: https://www.mdpi.com/article/10.3390/ani15233484/s1, Table S1: Analysis of differences in cattle foraging plant composition and nutrient intake across various plots in undegraded Grassland; Table S2: Analysis of differences in cattle foraging plant composition and nutrient intake across various plots in severe saline-alkaline degraded grassland; Table S3: Summary of linear mixed effects models analyzing the effects of nutrient intake and foraging plant species diversity to the relative abundance of the key bacteria in feces. Mineral element intake and simpson index were taken as fixed factors; grassland types (undegraded grassland and severe saline-alkaline degraded grassland) were taken as random factors. Factors that only have significant effect on key bacteria are presented in the table; Table S4: Summary of linear mixed models analyzing the relative importance of various mineral elements and foraging plant species diversity for key fecal bacteria. Mineral element intake and foraging plant simpson index were taken as fixed factors; grassland types (undegraded grassland and severe saline-alkaline degraded grassland) were taken as random factors; Text S1: The calculation module of functional indices.
