# Supplementary material for: Grassland Saline-Alkaline Degradation-Induced Excessive Iron and Sodium Intake Potentially Increases the Transmission Risk of Fecal Pathogenic Bacteria in Cattle
Source: Animals (Basel). 2025 Dec 3;15(23):3484. doi: 10.3390/ani15233484 (PMC12691539; doi:10.3390/ani15233484)
Supplement: Supplementary file 1 [file animals-15-03484-s001.zip › animals-3980918-supplementary.pdf]

Supplementary Tables

Table S1 Analysis of differences in cattle foraging plant composition and nutrient intake across various plots in undegraded Grassland.

Item	UG1	UG2	UG3	F-value	*p*-value
Plant composition (%)					
Leymus chinensis	5.18 ± 0.24	5.15 ± 0.18	5.96 ± 1.00	0.58	0.59
Phragmites australis	3.22 ± 0.09	3.57 ± 0.15	4.27 ± 0.41	4.38	0.07
Kalimeris indica	0.50 ± 0.02	0.35 ± 0.13	1.05 ± 0.62	1.03	0.41
Calamagrostis epigeios	46.32 ± 0.73	46.4 ± 1.34	46.1 ± 0.14	0.03	0.97
Carex duriuscula	2.45 ± 0.10	2.42 ± 0.33	2.84 ± 0.59	0.36	0.72
Tournefortia sibirica	0.61 ± 0.01	0.96 ± 0.12	1.16 ± 0.62	0.59	0.58
Chloris virgata	0.89 ± 0.02	0.71 ± 0.14	1.43 ± 0.62	1.06	0.40
Artemisia scoparia	9.88 ± 0.49	10.41 ± 0.35	7.95 ± 0.33	0.86	0.47
Artemisia mongolica	1.96 ± 0.11	1.73 ± 0.13	2.42 ± 0.61	0.93	0.45
Knorringia sibirica	3.73 ± 0.08	3.59 ± 0.10	4.25 ± 0.57	1.08	0.40
Lespedeza davurica	13.14 ± 0.23	13.06 ± 0.17	9.98 ± 3.23	0.91	0.45
Lespedeza chinensis	12.14 ± 0.51	11.64 ± 0.16	12.57 ± 0.49	1.28	0.35
Nutrient intake					
DM (kg/d)	13.97 ± 1.13	15.93 ± 0.10	16.70 ± 1.13	2.33	0.18
NDF (kg/d)	867.86 ± 69.77	989.22 ± 7.69	1043.01 ± 68.71	2.51	0.16
ADF (kg/d)	512.04 ± 41.72	583.50 ± 2.96	612.34 ± 40.91	2.34	0.18
CP (kg/d)	132.55 ± 11.01	151.51 ± 0.73	156.47 ± 11.58	1.86	0.24
EE (kg/d)	119.4 ± 9.54	137.40 ± 1.18	144.98 ± 9.35	2.87	0.13
OM (kg/d)	1294.55 ± 105.05	1476.45 ± 9.29	1548.73 ± 104.19	2.34	0.18
Fe (mg/d)	1958.54 ± 161.61	2225.20 ± 10.13	2339.49 ± 158.40	2.23	0.19
Cu (mg/d)	59.85 ± 5.14	67.89 ± 0.43	70.22 ± 5.28	1.63	0.27
Mn (mg/d)	435.47 ± 35.23	495.97 ± 2.46	524.84 ± 33.86	2.61	0.15
Zn (mg/d)	269.04 ± 22.34	305.18 ± 1.38	320.59 ± 21.59	2.17	0.20
Ca (g/d)	67.33 ± 5.76	76.50 ± 0.16	77.00 ± 7.24	1.04	0.41
K (g/d)	132.68 ± 10.87	151.17 ± 0.73	159.47 ± 10.60	2.45	0.17
Na (g/d)	23.66 ± 2.14	26.33 ± 0.47	28.46 ± 1.80	2.16	0.20
Mg (g/d)	20.91 ± 1.75	23.65 ± 0.14	24.84 ± 1.70	2.04	0.21

Table S2 Analysis of differences in cattle foraging plant composition and nutrient intake across various plots in severe saline-alkaline degraded grassland

Item	SG1	SG2	SG3	F-value	*p*-value
Plant composition (%)					
Leymus chinensis	8.91 ± 0.08	8.68 ± 0.24	9.06 ± 0.03	1.76	0.25
Phragmites australis	10.79 ± 0.41	11.02 ± 0.57	10.31 ± 0.01	0.80	0.49
Kalimeris indica	3.58 ± 0.04	3.49 ± 0.19	3.67 ± 0.03	0.62	0.57
Carex duriuscula	0.34 ± 0.04	0.24 ± 0.15	0.38 ± 0.05	0.52	0.62
Chloris virgata	1.07 ± 0.11	0.98 ±0.16	1.10 ± 0.05	0.30	0.75
Artemisia scoparia	0.91 ± 0.32	1.00 ± 0.27	0.38 ± 0.05	1.93	0.23
Kochia scoparia	74.06 ± 0.38	74.33 ± 0.19	74.71 ± 0.24	1.43	0.31
Artemisia mongolica	0.35 ± 0.05	0.26 ± 0.16	0.39 ± 0.06	0.40	0.69
Nutrient intake					
DM (kg/d)	14.55 ± 0.20	15.10 ± 0.23	14.81 ± 0.41	0.89	0.46
NDF (kg/d)	695.12 ± 8.95	720.85 ± 10.88	706.66 ± 20.22	0.82	0.48
ADF (kg/d)	404.61 ± 5.04	419.6 ± 6.79	411.02 ± 11.75	0.81	0.49
CP (kg/d)	162.34 ± 2.18	168.77 ± 2.70	165.22 ± 4.48	0.98	0.43
EE (kg/d)	142.98 ± 1.77	148.60 ± 2.63	145.32 ± 3.95	0.94	0.44
OM (kg/d)	1217.73 ± 16.02	1263.83 ± 19.98	1238.85 ± 34.64	0.88	0.46
Fe (mg/d)	3812.77 ± 60.48	3962.13 ± 57.60	3899.34 ± 103.04	0.96	0.43
Cu (mg/d)	78.88 ± 1.12	81.85 ± 1.53	80.42 ± 2.17	0.8	0.49
Mn (mg/d)	613.24 ± 8.23	637.68 ± 9.20	624.12 ± 17.06	1.01	0.42
Zn (mg/d)	304.28 ± 4.33	315.75 ± 4.94	310.36 ± 8.50	0.86	0.47
Ca (g/d)	65.38 ± 0.87	67.94 ± 1.32	66.47 ± 1.79	0.87	0.47
K (g/d)	150.9 ± 2.11	156.52 ± 2.43	153.73 ± 4.30	0.82	0.49
Na (g/d)	403.08 ± 7.22	419.73 ± 5.42	413.8 ± 10.36	1.13	0.38
Mg (g/d)	32.74 ± 0.49	34.02 ± 0.47	33.46 ± 0.89	0.98	0.43

Table S3. Summary of linear mixed effects models analyzing the effects of nutrient intake and foraging plant species diversity to the relative abundance of the key bacteria in feces. Mineral element intake and simpson index were taken as fixed factors; grassland types (undegraded grassland and severe saline-alkaline degraded grassland) were taken as random factors. Factors that only have significant effect on key bacteria are presented in the table.

Key bacteria	Variable	DF	F-value	*p*-value
Paludibacter	Fe	5	214.68	<0.01
	Na	5	39.82	<0.01
	Cu	5	9.71	0.03
	Zn	5	7.90	0.04
	Mg	5	22.39	<0.01
	Simpson index	5	7.19	0.04
Streptococcus	Fe	5	47.07	<0.01
	Na	5	25.53	<0.01
	Cu	5	12.91	0.02
	Mn	5	35.07	<0.01
	Mg	5	31.90	<0.01
	Simpson index	5	17.60	<0.01
Alistipes	Na	5	65.01	<0.01
	Cu	5	11.23	0.02
	Mg	5	9.26	0.03
	Simpson index	5	16.14	0.01

Table S4. Summary of linear mixed models analyzing the relative importance of various mineral elements and foraging plant species diversity for key fecal bacteria. Mineral element intake and foraging plant simpson index were taken as fixed factors; grassland types (undegraded grassland and severe saline-alkaline degraded grassland) were taken as random factors.

Key bacteria	Variable	DF	F-value	*p*-value
Paludibacter	Fe	4	38.69	0.06
	Na	4	6.10	0.10
	Fe	4	196.67	<0.01
	Cu	4	0.50	0.52
	Fe	4	200.71	<0.01
	Zn	4	0.61	0.48
	Fe	4	249.54	0.05
	Mg	4	1.97	0.23
	Fe	4	260.34	<0.01
	Simpson index	4	2.28	0.21
Streptococcus	Fe	4	38.63	<0.01
	Na	4	0.29	0.61
	Fe	4	51.08	<0.01
	Cu	4	1.04	0.37
	Fe	4	38.14	0.02
	Mn	4	0.07	0.81
	Fe	4	54.1	<0.01
	Mg	4	1.53	0.28
	Fe	4	38.12	<0.01
	Simpson index	4	0.04	0.84
Alistipes	Na	4	177.87	<0.01
	Cu	4	9.19	0.11
	Na	4	115.61	<0.01
	Mg	4	4.51	0.10
	Na	4	61.85	0.02
	Simpson index	4	0.49	0.52

Supplementary Text S1

The calculation module of functional indices

```
abu=read.csv("zkwab.csv",header = T)
library(spaa)
ck.mat<- data2mat(abu)
ck.mat
write.csv(ck.mat,"zkwab1.csv")
abu2=read.csv("zkwab1.csv",row.names = 1)
trait=read.csv("zkwtrait.csv",row.names = 1)
fd <- dbFD(trait, abu2)
fd write.csv(fd, "zkwcwm.csv")
library(FD)
```

Detailed parameters of linear mixed effects models

The equation of model is $Y_{ij} = X_{ij}\beta + Z_{ij}\mu_i + \epsilon_{ij}$, where Y_{ij} is the observed response for the *j-th* observation within group *i*, X_{ij} is a vector of covariates associated with the fixed effects for the *j-th* observation in group *i*, β is the population mean which represents fixed-effect factors, Z_{ij} is a vector of variables associated with the random effects for the *j-th* observation in group *i*, μ_i is intercepts relative to population mean across different groups which means variability between groups, ϵ_{ij} is the error term for the *j-th* observation in group i representing the unexplained variability within the group. Thus, $X_{ij}\beta$ captures fixed effects, $Z_{ij}\mu_j$ captures random effects, and ϵ_{ij} means random error. The random effect structure was specified as a random intercept model.
